# (μ_3_-Hydrido)[μ_3_-2-(tri­methyl­sil­yl)ethyl­idyne-κ^3^ *C* ^1^:*C* ^1^:*C* ^1^]tetra­kis­[(η^5^-cyclo­penta­dien­yl)cobalt(II)]

**DOI:** 10.1107/S1600536813030432

**Published:** 2013-11-09

**Authors:** Martin Haehnel, Anke Spannenberg, Uwe Rosenthal

**Affiliations:** a Leibniz-Institut für Katalyse e. V. an der Universität Rostock, Albert-Einstein-Strasse 29a, 18059 Rostock, Germany

## Abstract

In the title compound, [Co_4_(C_5_H_5_)_4_(μ_3_-CCH_2_SiMe_3_)(μ_3_-H)], the Co atoms form a distorted tetra­hedron with the ethyl­idyne moiety bridging three of the Co atoms as well as the hydrido ligand also bridging three of the Co atoms. The Co—Co bond lengths in the Co_4_ tetrahedron vary from 2.3844 (4) to 2.4608 (4) Å. Each Co atom is additionally η^5^-bonded to a cyclopentadienyl (Cp) anion.

## Related literature

For other tetra­nuclear Co clusters with a tetra­hedral Co_4_ core featuring μ_3_-bridging hydrido ligands, see: Huttner & Lorenz (1975 ▶); Stella *et al.* (1988 ▶); Wadepohl & Pritzkow (1993 ▶); Schneider *et al.* (1997 ▶); Bau *et al.* (2004 ▶). [CpCo]_4_ clusters with μ_3_-bridging carbonyl groups are described by Gambarotta *et al.* (1985 ▶) and Stella *et al.* (1988 ▶). For [CpCo]_4_ clusters with μ_3_-bridging hydrido and μ_3_-bridging C—CH_3_ ligands, see: Stella *et al.* (1988 ▶) and Wadepohl & Pritzkow (1993 ▶). The starting alkyne complex Cp*_2_Ti(η^2^-Ph_2_PC_2_PPh_2_) is described by Haehnel *et al.* (2013 ▶). For the starting Co complex CpCo(H_2_C=CHSiMe_3_)_2_, see: Hapke *et al.* (2010 ▶).
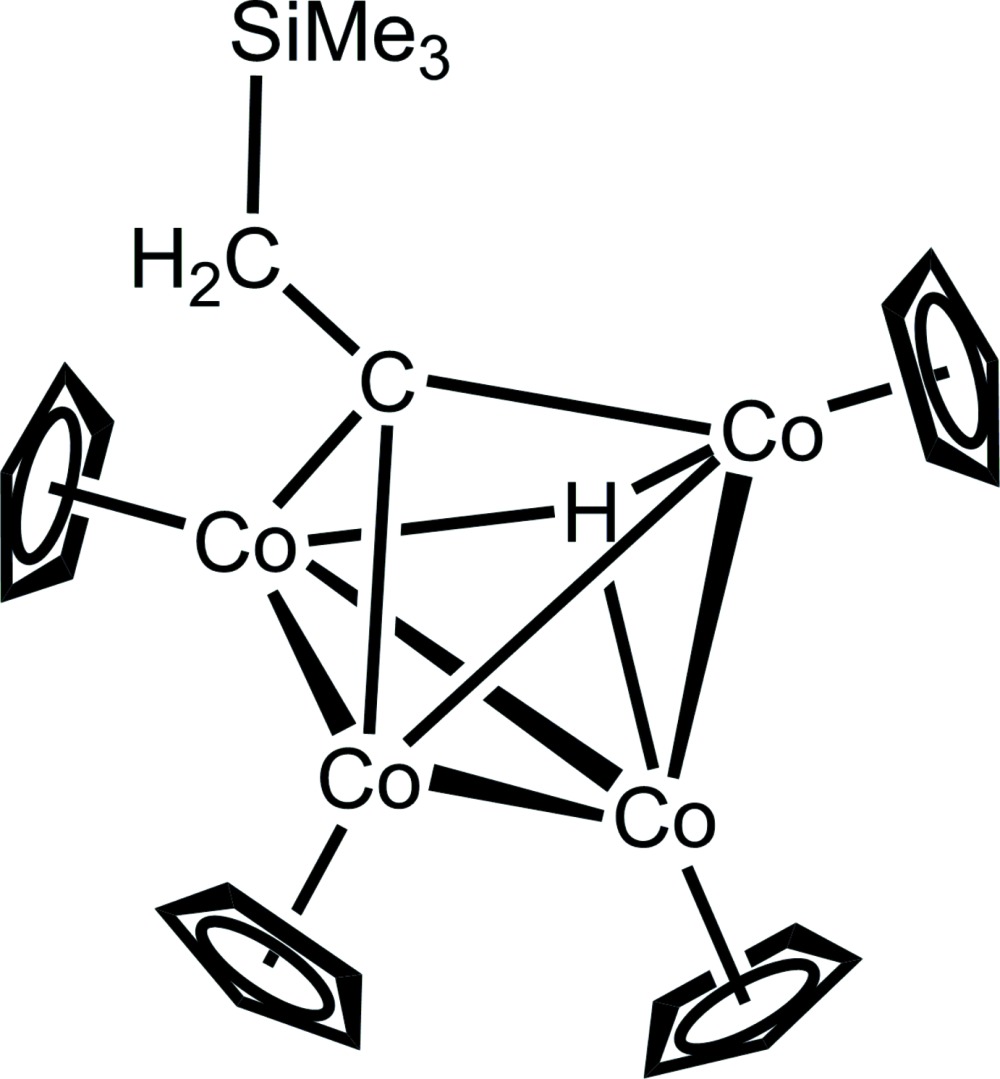


## Experimental

### 

#### Crystal data

[Co_4_(C_5_H_5_)_4_(C_5_H_11_Si)H]
*M* *_r_* = 596.32
Monoclinic, 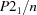

*a* = 9.3691 (4) Å
*b* = 17.7016 (8) Å
*c* = 14.2208 (7) Å
β = 92.779 (3)°
*V* = 2355.72 (19) Å^3^
*Z* = 4
Mo *K*α radiation
μ = 2.83 mm^−1^
*T* = 150 K
0.27 × 0.14 × 0.04 mm

#### Data collection

Bruker Kappa APEXII DUO diffractometer
Absorption correction: multi-scan (*SADABS*; Bruker, 2008 ▶) *T*_min_ = 0.891, *T*_max_ = 1.000
62865 measured reflections
5682 independent reflections
4481 reflections with *I* > 2σ(*I*)
*R*_int_ = 0.066

#### Refinement

*R*[*F* ^2^ > 2σ(*F* ^2^)] = 0.028
*wR*(*F* ^2^) = 0.058
*S* = 1.03
5682 reflections
262 parameters
H atoms treated by a mixture of independent and constrained refinement
Δρ_max_ = 0.52 e Å^−3^
Δρ_min_ = −0.32 e Å^−3^

### 

Data collection: *APEX2* (Bruker, 2011 ▶); cell refinement: *SAINT* (Bruker, 2009 ▶); data reduction: *SAINT*; program(s) used to solve structure: *SHELXS97* (Sheldrick, 2008 ▶); program(s) used to refine structure: *SHELXL97* (Sheldrick, 2008 ▶); molecular graphics: *XP* in *SHELXTL* (Sheldrick, 2008 ▶); software used to prepare material for publication: *SHELXL97*.

## Supplementary Material

Crystal structure: contains datablock(s) I, Global. DOI: 10.1107/S1600536813030432/pk2502sup1.cif

Structure factors: contains datablock(s) I. DOI: 10.1107/S1600536813030432/pk2502Isup2.hkl

Additional supplementary materials:  crystallographic information; 3D view; checkCIF report

## Figures and Tables

**Fig. 1.**
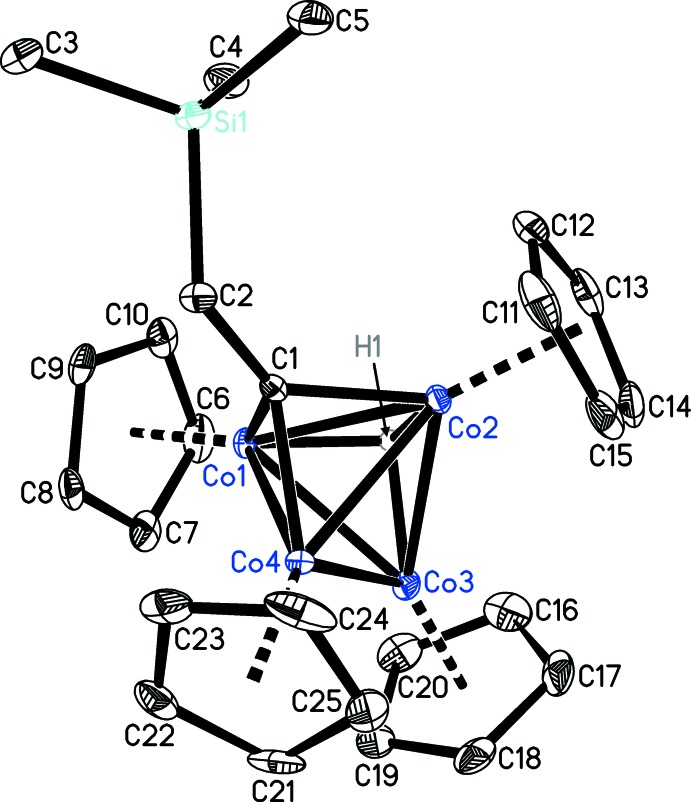
Molecular structure of the title compound in the crystal. Hydrogen atoms except H1 are omitted for clarity. Displacement ellipsoids are drawn at the 30% probability level.

## supplementary crystallographic information

### 1. Comment

The reaction of the [CpCo] precursor CpCo(hexadiene) with the titanocene alkyne complex Cp*_2_Ti(η^2^-Ph_2_PC_2_PPh_2_) was investigated to synthesize a new heterobimetallic complex. However, only the degradation of the [CpCo] precursor complex was observed. The title compound consists of a tetrahedral Co_4_ core. Two Co atoms (Co1 and Co2) feature a hexa-coordination by one Cp ligand, three Co atoms and the bridging hydrido- and ethylidyne unit. The other two Co atoms (Co3 and Co4) feature a penta-coordination mode. While the Co3 atom is surrounded by the Cp unit, three Co atoms and the hydrido ligand, the Co4 atom is coordinated by its Cp ligand, also three Co atoms and the ethylidyne unit. The Co—Co distances vary from 2.3844 (4) Å (Co3—Co4) to 2.4608 (4) Å (Co1—Co2). The vector C1-C2 is almost perpendicular to the Co1, Co2, Co4 plane and the Co—C distances are similar to those obtained for a Co_4_ cluster described by Wadepohl *et al.* (1993). The µ_3_-bridging hydride was found in a difference Fourier map and Co—H distances of about 1.65 Å were found (Co1—H1 1.67 (3), Co2—H1 1.64 (3), Co3—H1 1.65 (3) Å).

### 2. Experimental

To a stirred solution of Cp*_2_Ti(η^2^-Ph_2_PC_2_PPh_2_) (180 mg, 0.253 mmol) in 10 ml of THF was added a solution of crude CpCo(hexadiene) (0.81 *M*, 3.12 ml) (contaminated with a small amount of CpCo(H_2_C=CHSiMe_3_)_2_) in THF. The reaction mixture was stirred for 16 h at 45°C and then cooled to room temperature. After removing all the volatiles in vacuum, the dark brown residue was dissolved in 6 ml of a mixture of THF/*n*-hexane (1:2). Dark brown crystals of the title compound, suitable for X-ray analysis were grown after 14 days at -78 °C, being a degradation product of the small impurities mentioned above. Noteworthy, the µ_3_-(trimethylsilyl)ethylidyne unit results from this starting material CpCo(H_2_C=CHSiMe_3_)_2_.

### 3. Refinement

The hydride was found in a difference Fourier map and was refined freely. All other H atoms were placed at idealized positions with d(C—H) = 0.95 Å (CH), 0.99 Å (CH_2_) and 0.98 Å (CH_3_) and refined using a riding model with *U*_iso_(H) fixed at 1.2 *U*_eq_(C) for CH, CH_2_ and 1.5 *U*_eq_(C) for CH_3_.

### Crystal data

**Table:**

[Co_4_(C_5_H_5_)_4_(C_5_H_11_Si)H]	*F*(000) = 1216
*M**_r_* = 596.32	*D*_x_ = 1.681 Mg m^−^^3^
Monoclinic, *P*2_1_/*n*	Mo *K*α radiation, λ = 0.71073 Å
*a* = 9.3691 (4) Å	Cell parameters from 9904 reflections
*b* = 17.7016 (8) Å	θ = 2.3–27.2°
*c* = 14.2208 (7) Å	µ = 2.83 mm^−^^1^
β = 92.779 (3)°	*T* = 150 K
*V* = 2355.72 (19) Å^3^	Plate, brown
*Z* = 4	0.27 × 0.14 × 0.04 mm

### Data collection

**Table:**

Bruker Kappa APEXII DUO diffractometer	5682 independent reflections
Radiation source: fine-focus sealed tube	4481 reflections with *I* > 2σ(*I*)
Curved graphite monochromator	*R*_int_ = 0.066
Detector resolution: 8.3333 pixels mm^-1^	θ_max_ = 28.0°, θ_min_ = 1.8°
φ and ω scans	*h* = −12→12
Absorption correction: multi-scan (*SADABS*; Bruker, 2008)	*k* = −23→23
*T*_min_ = 0.891, *T*_max_ = 1.000	*l* = −18→18
62865 measured reflections	

### Refinement

**Table:**

Refinement on *F*^2^	Primary atom site location: structure-invariant direct methods
Least-squares matrix: full	Secondary atom site location: difference Fourier map
*R*[*F*^2^ > 2σ(*F*^2^)] = 0.028	Hydrogen site location: inferred from neighbouring sites
*wR*(*F*^2^) = 0.058	H atoms treated by a mixture of independent and constrained refinement
*S* = 1.03	*w* = 1/[σ^2^(*F*_o_^2^) + (0.0181*P*)^2^ + 2.1633*P*] where *P* = (*F*_o_^2^ + 2*F*_c_^2^)/3
5682 reflections	(Δ/σ)_max_ < 0.001
262 parameters	Δρ_max_ = 0.52 e Å^−^^3^
0 restraints	Δρ_min_ = −0.32 e Å^−^^3^

### Special details

**Table:**

Geometry. All e.s.d.'s (except the e.s.d. in the dihedral angle between two l.s. planes) are estimated using the full covariance matrix. The cell e.s.d.'s are taken into account individually in the estimation of e.s.d.'s in distances, angles and torsion angles; correlations between e.s.d.'s in cell parameters are only used when they are defined by crystal symmetry. An approximate (isotropic) treatment of cell e.s.d.'s is used for estimating e.s.d.'s involving l.s. planes.
Refinement. Refinement of *F*^2^ against ALL reflections. The weighted *R*-factor *wR* and goodness of fit *S* are based on *F*^2^, conventional *R*-factors *R* are based on *F*, with *F* set to zero for negative *F*^2^. The threshold expression of *F*^2^ > σ(*F*^2^) is used only for calculating *R*-factors(gt) *etc*. and is not relevant to the choice of reflections for refinement. *R*-factors based on *F*^2^ are statistically about twice as large as those based on *F*, and *R*- factors based on ALL data will be even larger.

### Fractional atomic coordinates and isotropic or equivalent isotropic displacement parameters (Å^2^)

**Table:**

	*x*	*y*	*z*	*U*_iso_*/*U*_eq_	
C1	0.4449 (2)	0.10065 (12)	0.25656 (15)	0.0144 (4)	
C2	0.5227 (2)	0.07279 (14)	0.34492 (16)	0.0200 (5)	
H2A	0.4492	0.0514	0.3850	0.024*	
H2B	0.5614	0.1181	0.3781	0.024*	
C3	0.8023 (3)	0.02862 (17)	0.4434 (2)	0.0358 (7)	
H3A	0.7523	0.0312	0.5022	0.054*	
H3B	0.8436	0.0781	0.4301	0.054*	
H3C	0.8788	−0.0091	0.4495	0.054*	
C4	0.7712 (3)	−0.00180 (16)	0.2340 (2)	0.0329 (6)	
H4A	0.8323	−0.0467	0.2342	0.049*	
H4B	0.8303	0.0436	0.2295	0.049*	
H4C	0.7023	−0.0040	0.1800	0.049*	
C5	0.6069 (3)	−0.09495 (15)	0.3726 (2)	0.0328 (6)	
H5A	0.6882	−0.1291	0.3843	0.049*	
H5B	0.5469	−0.1138	0.3193	0.049*	
H5C	0.5504	−0.0927	0.4288	0.049*	
C6	0.6245 (3)	0.22244 (16)	0.06694 (18)	0.0319 (6)	
H6	0.6135	0.2184	0.0004	0.038*	
C7	0.5565 (3)	0.27580 (15)	0.12302 (19)	0.0303 (6)	
H7	0.4925	0.3142	0.1010	0.036*	
C8	0.6004 (3)	0.26194 (15)	0.21753 (19)	0.0275 (6)	
H8	0.5706	0.2892	0.2707	0.033*	
C9	0.6966 (3)	0.20041 (16)	0.21950 (18)	0.0265 (6)	
H9	0.7431	0.1791	0.2741	0.032*	
C10	0.7113 (3)	0.17631 (16)	0.12609 (18)	0.0278 (6)	
H10	0.7697	0.1359	0.1065	0.033*	
C11	0.2899 (3)	−0.04602 (12)	0.21512 (14)	0.0433 (8)	
H11	0.2953	−0.0545	0.2812	0.052*	
C12	0.3974 (2)	−0.06058 (11)	0.15344 (15)	0.0359 (7)	
H12	0.4891	−0.0808	0.1700	0.043*	
C13	0.3469 (2)	−0.04038 (11)	0.06330 (14)	0.0374 (7)	
H13	0.3981	−0.0444	0.0075	0.045*	
C14	0.2081 (2)	−0.01335 (12)	0.06928 (17)	0.0457 (8)	
H14	0.1480	0.0044	0.0182	0.055*	
C15	0.1729 (2)	−0.01683 (12)	0.16311 (19)	0.0533 (11)	
H15	0.0845	−0.0019	0.1874	0.064*	
C16	0.2190 (4)	0.17467 (19)	−0.0635 (2)	0.0463 (8)	
H16	0.2581	0.1415	−0.1080	0.056*	
C17	0.0924 (3)	0.16256 (18)	−0.0154 (2)	0.0438 (8)	
H17	0.0313	0.1199	−0.0221	0.053*	
C18	0.0727 (3)	0.22448 (17)	0.0441 (2)	0.0341 (6)	
H18	−0.0040	0.2311	0.0846	0.041*	
C19	0.1864 (3)	0.27501 (16)	0.03311 (19)	0.0321 (6)	
H19	0.2004	0.3217	0.0652	0.038*	
C20	0.2762 (3)	0.24413 (18)	−0.03388 (19)	0.0368 (7)	
H20	0.3609	0.2666	−0.0552	0.044*	
C21	0.1300 (2)	0.24468 (11)	0.27716 (14)	0.0361 (7)	
H21	0.0887	0.2789	0.2321	0.043*	
C22	0.2522 (2)	0.25765 (11)	0.33478 (14)	0.0330 (6)	
H22	0.3087	0.3022	0.3359	0.040*	
C23	0.2769 (2)	0.19371 (12)	0.39046 (13)	0.0368 (7)	
H23	0.3532	0.1871	0.4362	0.044*	
C24	0.1700 (2)	0.14122 (11)	0.36725 (14)	0.0426 (8)	
H24	0.1606	0.0925	0.3944	0.051*	
C25	0.07921 (19)	0.17272 (12)	0.29723 (15)	0.0404 (8)	
H25	−0.0029	0.1493	0.2683	0.049*	
Co1	0.49716 (3)	0.167531 (17)	0.16344 (2)	0.01454 (7)	
Co2	0.34418 (3)	0.054480 (17)	0.15636 (2)	0.01542 (7)	
Co3	0.26521 (3)	0.172517 (18)	0.08131 (2)	0.01868 (8)	
Co4	0.28421 (3)	0.162137 (17)	0.24862 (2)	0.01396 (7)	
Si1	0.67352 (7)	0.00134 (4)	0.34501 (5)	0.02116 (14)	
H1	0.406 (3)	0.1162 (16)	0.0821 (19)	0.034 (8)*	

### Atomic displacement parameters (Å^2^)

**Table:**

	*U*^11^	*U*^22^	*U*^33^	*U*^12^	*U*^13^	*U*^23^
C1	0.0154 (11)	0.0123 (11)	0.0158 (10)	−0.0006 (9)	0.0036 (9)	−0.0012 (8)
C2	0.0209 (12)	0.0210 (12)	0.0181 (11)	0.0046 (10)	0.0022 (9)	0.0015 (9)
C3	0.0307 (15)	0.0318 (15)	0.0434 (17)	0.0071 (13)	−0.0144 (13)	0.0018 (13)
C4	0.0268 (14)	0.0311 (15)	0.0414 (16)	0.0075 (12)	0.0097 (12)	−0.0025 (13)
C5	0.0322 (15)	0.0219 (13)	0.0438 (16)	0.0047 (12)	−0.0036 (13)	0.0063 (12)
C6	0.0329 (15)	0.0415 (17)	0.0220 (13)	−0.0215 (13)	0.0078 (11)	0.0032 (12)
C7	0.0296 (14)	0.0212 (13)	0.0394 (15)	−0.0146 (11)	−0.0034 (12)	0.0078 (12)
C8	0.0259 (13)	0.0255 (14)	0.0312 (14)	−0.0133 (11)	0.0025 (11)	−0.0092 (11)
C9	0.0168 (12)	0.0350 (15)	0.0275 (13)	−0.0114 (11)	−0.0010 (10)	−0.0026 (11)
C10	0.0188 (12)	0.0326 (15)	0.0328 (14)	−0.0112 (11)	0.0093 (11)	−0.0054 (12)
C11	0.071 (2)	0.0227 (15)	0.0366 (16)	−0.0214 (15)	0.0100 (16)	0.0003 (13)
C12	0.0317 (15)	0.0153 (13)	0.060 (2)	−0.0001 (11)	−0.0059 (14)	−0.0029 (13)
C13	0.0504 (18)	0.0235 (15)	0.0390 (16)	−0.0131 (13)	0.0091 (14)	−0.0161 (12)
C14	0.0482 (19)	0.0232 (15)	0.063 (2)	−0.0102 (14)	−0.0300 (17)	−0.0059 (14)
C15	0.0242 (15)	0.0275 (16)	0.111 (3)	−0.0152 (13)	0.0277 (18)	−0.0272 (19)
C16	0.069 (2)	0.049 (2)	0.0193 (13)	0.0227 (18)	−0.0176 (14)	−0.0030 (13)
C17	0.0483 (19)	0.0318 (16)	0.0479 (18)	−0.0049 (14)	−0.0329 (15)	0.0054 (14)
C18	0.0261 (14)	0.0368 (16)	0.0383 (16)	0.0044 (12)	−0.0096 (12)	0.0136 (13)
C19	0.0368 (15)	0.0255 (14)	0.0329 (14)	0.0052 (12)	−0.0089 (12)	0.0071 (12)
C20	0.0414 (16)	0.0437 (18)	0.0248 (14)	0.0050 (14)	−0.0034 (12)	0.0179 (13)
C21	0.0432 (17)	0.0350 (16)	0.0306 (15)	0.0294 (14)	0.0071 (13)	0.0022 (12)
C22	0.0358 (15)	0.0259 (14)	0.0389 (16)	0.0019 (12)	0.0161 (13)	−0.0128 (12)
C23	0.0399 (16)	0.0522 (19)	0.0182 (13)	0.0254 (15)	0.0024 (12)	−0.0103 (12)
C24	0.061 (2)	0.0259 (15)	0.0446 (18)	0.0168 (15)	0.0406 (16)	0.0113 (13)
C25	0.0162 (13)	0.0483 (19)	0.058 (2)	−0.0015 (13)	0.0131 (13)	−0.0214 (16)
Co1	0.01496 (15)	0.01619 (15)	0.01250 (14)	−0.00413 (13)	0.00114 (11)	−0.00058 (12)
Co2	0.01599 (15)	0.01296 (15)	0.01731 (15)	−0.00182 (12)	0.00085 (11)	−0.00215 (12)
Co3	0.02114 (16)	0.01829 (16)	0.01602 (15)	−0.00056 (13)	−0.00513 (12)	0.00215 (13)
Co4	0.01403 (15)	0.01336 (15)	0.01463 (14)	0.00218 (12)	0.00197 (11)	0.00088 (12)
Si1	0.0183 (3)	0.0192 (3)	0.0257 (3)	0.0042 (3)	−0.0019 (3)	0.0018 (3)

### Geometric parameters (Å, º)

**Table:**

C1—C2	1.505 (3)	C14—C15	1.3914 (15)
C1—Co4	1.857 (2)	C14—Co2	2.109 (2)
C1—Co1	1.860 (2)	C14—H14	0.9500
C1—Co2	1.860 (2)	C15—Co2	2.047 (2)
C2—Si1	1.896 (2)	C15—H15	0.9500
C2—H2A	0.9900	C16—C20	1.398 (4)
C2—H2B	0.9900	C16—C17	1.415 (5)
C3—Si1	1.866 (3)	C16—Co3	2.084 (3)
C3—H3A	0.9800	C16—H16	0.9500
C3—H3B	0.9800	C17—C18	1.402 (4)
C3—H3C	0.9800	C17—Co3	2.080 (3)
C4—Si1	1.863 (3)	C17—H17	0.9500
C4—H4A	0.9800	C18—C19	1.406 (4)
C4—H4B	0.9800	C18—Co3	2.071 (3)
C4—H4C	0.9800	C18—H18	0.9500
C5—Si1	1.864 (3)	C19—C20	1.411 (4)
C5—H5A	0.9800	C19—Co3	2.063 (3)
C5—H5B	0.9800	C19—H19	0.9500
C5—H5C	0.9800	C20—Co3	2.078 (3)
C6—C10	1.403 (4)	C20—H20	0.9500
C6—C7	1.408 (4)	C21—C22	1.3942 (14)
C6—Co1	2.100 (2)	C21—C25	1.3942 (14)
C6—H6	0.9500	C21—Co4	2.1078 (18)
C7—C8	1.408 (4)	C21—H21	0.9500
C7—Co1	2.084 (2)	C22—C23	1.3942 (14)
C7—H7	0.9500	C22—Co4	2.1178 (19)
C8—C9	1.413 (4)	C22—H22	0.9500
C8—Co1	2.061 (2)	C23—C24	1.3942 (14)
C8—H8	0.9500	C23—Co4	2.0973 (19)
C9—C10	1.408 (4)	C23—H23	0.9500
C9—Co1	2.079 (2)	C24—C25	1.3942 (14)
C9—H9	0.9500	C24—Co4	2.0745 (18)
C10—Co1	2.106 (2)	C24—H24	0.9500
C10—H10	0.9500	C25—Co4	2.0810 (18)
C11—C12	1.3914 (15)	C25—H25	0.9500
C11—C15	1.3914 (15)	Co1—Co4	2.3857 (4)
C11—Co2	2.040 (2)	Co1—Co3	2.4183 (4)
C11—H11	0.9500	Co1—Co2	2.4608 (4)
C12—C13	1.3914 (15)	Co1—H1	1.67 (3)
C12—Co2	2.098 (2)	Co2—Co4	2.3959 (4)
C12—H12	0.9500	Co2—Co3	2.4445 (4)
C13—C14	1.3914 (15)	Co2—H1	1.64 (3)
C13—Co2	2.139 (2)	Co3—Co4	2.3844 (4)
C13—H13	0.9500	Co3—H1	1.65 (3)
			
C2—C1—Co4	126.97 (15)	C7—Co1—C6	39.33 (11)
C2—C1—Co1	132.04 (16)	C1—Co1—C10	120.90 (10)
Co4—C1—Co1	79.87 (9)	C8—Co1—C10	66.33 (10)
C2—C1—Co2	134.67 (17)	C9—Co1—C10	39.32 (10)
Co4—C1—Co2	80.28 (9)	C7—Co1—C10	65.94 (11)
Co1—C1—Co2	82.85 (9)	C6—Co1—C10	38.98 (11)
C1—C2—Si1	123.36 (16)	C1—Co1—Co4	50.01 (7)
C1—C2—H2A	106.5	C8—Co1—Co4	103.41 (7)
Si1—C2—H2A	106.5	C9—Co1—Co4	125.09 (7)
C1—C2—H2B	106.5	C7—Co1—Co4	114.70 (8)
Si1—C2—H2B	106.5	C6—Co1—Co4	149.99 (9)
H2A—C2—H2B	106.5	C10—Co1—Co4	164.04 (7)
Si1—C3—H3A	109.5	C1—Co1—Co3	96.07 (7)
Si1—C3—H3B	109.5	C8—Co1—Co3	123.04 (8)
H3A—C3—H3B	109.5	C9—Co1—Co3	160.91 (8)
Si1—C3—H3C	109.5	C7—Co1—Co3	94.52 (8)
H3A—C3—H3C	109.5	C6—Co1—Co3	101.14 (8)
H3B—C3—H3C	109.5	C10—Co1—Co3	136.08 (8)
Si1—C4—H4A	109.5	Co4—Co1—Co3	59.510 (13)
Si1—C4—H4B	109.5	C1—Co1—Co2	48.58 (7)
H4A—C4—H4B	109.5	C8—Co1—Co2	159.48 (7)
Si1—C4—H4C	109.5	C9—Co1—Co2	138.96 (8)
H4A—C4—H4C	109.5	C7—Co1—Co2	154.06 (8)
H4B—C4—H4C	109.5	C6—Co1—Co2	134.26 (8)
Si1—C5—H5A	109.5	C10—Co1—Co2	127.58 (8)
Si1—C5—H5B	109.5	Co4—Co1—Co2	59.231 (12)
H5A—C5—H5B	109.5	Co3—Co1—Co2	60.127 (13)
Si1—C5—H5C	109.5	C1—Co1—H1	90.2 (9)
H5A—C5—H5C	109.5	C8—Co1—H1	156.0 (9)
H5B—C5—H5C	109.5	C9—Co1—H1	146.9 (9)
C10—C6—C7	108.4 (2)	C7—Co1—H1	116.3 (9)
C10—C6—Co1	70.73 (14)	C6—Co1—H1	95.0 (9)
C7—C6—Co1	69.73 (14)	C10—Co1—H1	109.2 (9)
C10—C6—H6	125.8	Co4—Co1—H1	85.2 (9)
C7—C6—H6	125.8	Co3—Co1—H1	42.9 (9)
Co1—C6—H6	125.3	Co2—Co1—H1	41.6 (9)
C8—C7—C6	107.7 (2)	C1—Co2—C11	101.35 (9)
C8—C7—Co1	69.27 (14)	C1—Co2—C15	127.21 (10)
C6—C7—Co1	70.94 (15)	C11—Co2—C15	39.8
C8—C7—H7	126.1	C1—Co2—C12	109.25 (9)
C6—C7—H7	126.1	C11—Co2—C12	39.3
Co1—C7—H7	125.2	C15—Co2—C12	65.78 (7)
C7—C8—C9	108.0 (2)	C1—Co2—C14	165.93 (9)
C7—C8—Co1	71.02 (14)	C11—Co2—C14	65.70 (7)
C9—C8—Co1	70.72 (14)	C15—Co2—C14	39.1
C7—C8—H8	126.0	C12—Co2—C14	64.72 (7)
C9—C8—H8	126.0	C1—Co2—C13	142.97 (9)
Co1—C8—H8	123.9	C11—Co2—C13	65.14 (7)
C10—C9—C8	107.8 (2)	C15—Co2—C13	65.02 (7)
C10—C9—Co1	71.37 (14)	C12—Co2—C13	38.3
C8—C9—Co1	69.37 (14)	C14—Co2—C13	38.2
C10—C9—H9	126.1	C1—Co2—Co4	49.81 (7)
C8—C9—H9	126.1	C11—Co2—Co4	113.54 (6)
Co1—C9—H9	124.8	C15—Co2—Co4	105.05 (6)
C6—C10—C9	108.0 (2)	C12—Co2—Co4	147.71 (6)
C6—C10—Co1	70.28 (14)	C14—Co2—Co4	128.56 (6)
C9—C10—Co1	69.31 (13)	C13—Co2—Co4	166.75 (6)
C6—C10—H10	126.0	C1—Co2—Co3	95.19 (7)
C9—C10—H10	126.0	C11—Co2—Co3	147.98 (7)
Co1—C10—H10	126.0	C15—Co2—Co3	109.04 (7)
C12—C11—C15	108.0	C12—Co2—Co3	152.68 (6)
C12—C11—Co2	72.59 (8)	C14—Co2—Co3	93.94 (6)
C15—C11—Co2	70.37 (8)	C13—Co2—Co3	114.41 (6)
C12—C11—H11	126.0	Co4—Co2—Co3	59.012 (13)
C15—C11—H11	126.0	C1—Co2—Co1	48.57 (7)
Co2—C11—H11	122.7	C11—Co2—Co1	148.06 (7)
C11—C12—C13	108.0	C15—Co2—Co1	162.88 (6)
C11—C12—Co2	68.15 (8)	C12—Co2—Co1	130.69 (6)
C13—C12—Co2	72.44 (8)	C14—Co2—Co1	145.22 (7)
C11—C12—H12	126.0	C13—Co2—Co1	129.75 (6)
C13—C12—H12	126.0	Co4—Co2—Co1	58.824 (12)
Co2—C12—H12	125.0	Co3—Co2—Co1	59.076 (13)
C12—C13—C14	108.0	C1—Co2—H1	91.1 (9)
C12—C13—Co2	69.23 (8)	C11—Co2—H1	160.9 (10)
C14—C13—Co2	69.70 (7)	C15—Co2—H1	137.7 (9)
C12—C13—H13	126.0	C12—Co2—H1	122.8 (9)
C14—C13—H13	126.0	C14—Co2—H1	102.8 (9)
Co2—C13—H13	126.6	C13—Co2—H1	96.3 (10)
C15—C14—C13	108.0	Co4—Co2—H1	85.5 (10)
C15—C14—Co2	68.08 (8)	Co3—Co2—H1	42.2 (9)
C13—C14—Co2	72.06 (7)	Co1—Co2—H1	42.6 (9)
C15—C14—H14	126.0	C19—Co3—C18	39.76 (11)
C13—C14—H14	126.0	C19—Co3—C20	39.85 (11)
Co2—C14—H14	125.4	C18—Co3—C20	66.64 (11)
C14—C15—C11	108.0	C19—Co3—C17	66.45 (12)
C14—C15—Co2	72.83 (8)	C18—Co3—C17	39.48 (12)
C11—C15—Co2	69.83 (8)	C20—Co3—C17	66.27 (12)
C14—C15—H15	126.0	C19—Co3—C16	66.54 (12)
C11—C15—H15	126.0	C18—Co3—C16	66.58 (12)
Co2—C15—H15	123.0	C20—Co3—C16	39.26 (12)
C20—C16—C17	107.8 (3)	C17—Co3—C16	39.71 (13)
C20—C16—Co3	70.12 (16)	C19—Co3—Co4	114.04 (8)
C17—C16—Co3	69.99 (16)	C18—Co3—Co4	108.14 (8)
C20—C16—H16	126.1	C20—Co3—Co4	146.05 (9)
C17—C16—H16	126.1	C17—Co3—Co4	132.14 (10)
Co3—C16—H16	125.4	C16—Co3—Co4	171.61 (11)
C18—C17—C16	108.1 (3)	C19—Co3—Co1	119.41 (8)
C18—C17—Co3	69.90 (15)	C18—Co3—Co1	153.00 (9)
C16—C17—Co3	70.30 (16)	C20—Co3—Co1	108.97 (9)
C18—C17—H17	125.9	C17—Co3—Co1	165.60 (10)
C16—C17—H17	125.9	C16—Co3—Co1	128.08 (10)
Co3—C17—H17	125.5	Co4—Co3—Co1	59.565 (12)
C17—C18—C19	107.9 (3)	C19—Co3—Co2	172.96 (8)
C17—C18—Co3	70.63 (16)	C18—Co3—Co2	137.12 (8)
C19—C18—Co3	69.83 (15)	C20—Co3—Co2	147.15 (9)
C17—C18—H18	126.0	C17—Co3—Co2	115.18 (9)
C19—C18—H18	126.0	C16—Co3—Co2	119.41 (9)
Co3—C18—H18	125.1	Co4—Co3—Co2	59.478 (12)
C18—C19—C20	108.0 (3)	Co1—Co3—Co2	60.797 (13)
C18—C19—Co3	70.41 (15)	C19—Co3—H1	143.7 (9)
C20—C19—Co3	70.63 (16)	C18—Co3—H1	162.6 (9)
C18—C19—H19	126.0	C20—Co3—H1	107.7 (9)
C20—C19—H19	126.0	C17—Co3—H1	123.2 (9)
Co3—C19—H19	124.6	C16—Co3—H1	98.3 (9)
C16—C20—C19	108.1 (3)	Co4—Co3—H1	85.8 (9)
C16—C20—Co3	70.62 (16)	Co1—Co3—H1	43.7 (9)
C19—C20—Co3	69.52 (15)	Co2—Co3—H1	42.0 (9)
C16—C20—H20	125.9	C1—Co4—C24	107.14 (9)
C19—C20—H20	125.9	C1—Co4—C25	142.31 (9)
Co3—C20—H20	125.5	C24—Co4—C25	39.2
C22—C21—C25	108.0	C1—Co4—C23	99.32 (8)
C22—C21—Co4	71.12 (7)	C24—Co4—C23	39.0
C25—C21—Co4	69.52 (7)	C25—Co4—C23	65.35 (6)
C22—C21—H21	126.0	C1—Co4—C21	162.91 (9)
C25—C21—H21	126.0	C24—Co4—C21	65.28 (6)
Co4—C21—H21	124.9	C25—Co4—C21	38.9
C21—C22—C23	108.0	C23—Co4—C21	64.88 (6)
C21—C22—Co4	70.35 (7)	C1—Co4—C22	124.74 (9)
C23—C22—Co4	69.89 (7)	C24—Co4—C22	65.10 (6)
C21—C22—H22	126.0	C25—Co4—C22	64.99 (6)
C23—C22—H22	126.0	C23—Co4—C22	38.6
Co4—C22—H22	125.3	C21—Co4—C22	38.5
C24—C23—C22	108.0	C1—Co4—Co3	97.29 (7)
C24—C23—Co4	69.59 (7)	C24—Co4—Co3	143.97 (7)
C22—C23—Co4	71.48 (7)	C25—Co4—Co3	107.40 (6)
C24—C23—H23	126.0	C23—Co4—Co3	159.19 (6)
C22—C23—H23	126.0	C21—Co4—Co3	96.89 (5)
Co4—C23—H23	124.5	C22—Co4—Co3	120.66 (6)
C23—C24—C25	108.0	C1—Co4—Co1	50.11 (7)
C23—C24—Co4	71.37 (7)	C24—Co4—Co1	153.79 (6)
C25—C24—Co4	70.65 (7)	C25—Co4—Co1	166.68 (6)
C23—C24—H24	126.0	C23—Co4—Co1	123.01 (6)
C25—C24—H24	126.0	C21—Co4—Co1	131.90 (6)
Co4—C24—H24	123.6	C22—Co4—Co1	114.02 (5)
C24—C25—C21	108.0	Co3—Co4—Co1	60.926 (13)
C24—C25—Co4	70.14 (7)	C1—Co4—Co2	49.91 (7)
C21—C25—Co4	71.60 (7)	C24—Co4—Co2	116.57 (6)
C24—C25—H25	126.0	C25—Co4—Co2	119.88 (6)
C21—C25—H25	126.0	C23—Co4—Co2	139.29 (6)
Co4—C25—H25	123.9	C21—Co4—Co2	146.92 (6)
C1—Co1—C8	112.67 (10)	C22—Co4—Co2	174.46 (6)
C1—Co1—C9	99.67 (10)	Co3—Co4—Co2	61.510 (13)
C8—Co1—C9	39.91 (10)	Co1—Co4—Co2	61.945 (12)
C1—Co1—C7	149.87 (10)	C4—Si1—C5	109.54 (13)
C8—Co1—C7	39.70 (10)	C4—Si1—C3	108.44 (14)
C9—Co1—C7	66.51 (11)	C5—Si1—C3	106.86 (13)
C1—Co1—C6	159.65 (11)	C4—Si1—C2	114.57 (12)
C8—Co1—C6	66.26 (10)	C5—Si1—C2	110.66 (12)
C9—Co1—C6	65.95 (10)	C3—Si1—C2	106.43 (12)
			
Co4—C1—C2—Si1	172.95 (12)	Co4—Co1—Co3—C19	102.20 (9)
Co1—C1—C2—Si1	−74.6 (3)	Co2—Co1—Co3—C19	171.93 (9)
Co2—C1—C2—Si1	56.4 (3)	C1—Co1—Co3—C18	104.69 (19)
C10—C6—C7—C8	−0.7 (3)	C8—Co1—Co3—C18	−17.4 (2)
Co1—C6—C7—C8	59.76 (17)	C9—Co1—Co3—C18	−40.7 (3)
C10—C6—C7—Co1	−60.43 (17)	C7—Co1—Co3—C18	−47.05 (19)
C6—C7—C8—C9	0.5 (3)	C6—Co1—Co3—C18	−86.2 (2)
Co1—C7—C8—C9	61.36 (17)	C10—Co1—Co3—C18	−106.6 (2)
C6—C7—C8—Co1	−60.82 (17)	Co4—Co1—Co3—C18	69.04 (18)
C7—C8—C9—C10	−0.2 (3)	Co2—Co1—Co3—C18	138.77 (18)
Co1—C8—C9—C10	61.34 (17)	C1—Co1—Co3—C20	−179.72 (11)
C7—C8—C9—Co1	−61.56 (17)	C8—Co1—Co3—C20	58.17 (13)
C7—C6—C10—C9	0.5 (3)	C9—Co1—Co3—C20	34.9 (2)
Co1—C6—C10—C9	−59.27 (17)	C7—Co1—Co3—C20	28.53 (12)
C7—C6—C10—Co1	59.80 (17)	C6—Co1—Co3—C20	−10.64 (13)
C8—C9—C10—C6	−0.2 (3)	C10—Co1—Co3—C20	−30.97 (15)
Co1—C9—C10—C6	59.87 (17)	Co4—Co1—Co3—C20	144.62 (9)
C8—C9—C10—Co1	−60.07 (16)	Co2—Co1—Co3—C20	−145.64 (9)
C15—C11—C12—C13	0.0	C1—Co1—Co3—C17	−111.6 (4)
Co2—C11—C12—C13	−61.83 (8)	C8—Co1—Co3—C17	126.3 (4)
C15—C11—C12—Co2	61.83 (8)	C9—Co1—Co3—C17	103.0 (4)
C11—C12—C13—C14	0.0	C7—Co1—Co3—C17	96.7 (4)
Co2—C12—C13—C14	−59.12 (8)	C6—Co1—Co3—C17	57.5 (4)
C11—C12—C13—Co2	59.12 (8)	C10—Co1—Co3—C17	37.2 (4)
C12—C13—C14—C15	0.0	Co4—Co1—Co3—C17	−147.2 (4)
Co2—C13—C14—C15	−58.82 (8)	Co2—Co1—Co3—C17	−77.5 (4)
C12—C13—C14—Co2	58.82 (8)	C1—Co1—Co3—C16	−140.14 (14)
C13—C14—C15—C11	0.0	C8—Co1—Co3—C16	97.75 (15)
Co2—C14—C15—C11	−61.33 (8)	C9—Co1—Co3—C16	74.4 (2)
C13—C14—C15—Co2	61.33 (8)	C7—Co1—Co3—C16	68.12 (14)
C12—C11—C15—C14	0.0	C6—Co1—Co3—C16	28.95 (15)
Co2—C11—C15—C14	63.26 (8)	C10—Co1—Co3—C16	8.61 (16)
C12—C11—C15—Co2	−63.26 (8)	Co4—Co1—Co3—C16	−175.79 (12)
C20—C16—C17—C18	−0.3 (3)	Co2—Co1—Co3—C16	−106.06 (12)
Co3—C16—C17—C18	59.89 (19)	C1—Co1—Co3—Co4	35.66 (7)
C20—C16—C17—Co3	−60.20 (19)	C8—Co1—Co3—Co4	−86.45 (9)
C16—C17—C18—C19	0.0 (3)	C9—Co1—Co3—Co4	−109.8 (2)
Co3—C17—C18—C19	60.14 (18)	C7—Co1—Co3—Co4	−116.09 (8)
C16—C17—C18—Co3	−60.14 (19)	C6—Co1—Co3—Co4	−155.26 (8)
C17—C18—C19—C20	0.3 (3)	C10—Co1—Co3—Co4	−175.60 (11)
Co3—C18—C19—C20	60.95 (18)	Co2—Co1—Co3—Co4	69.732 (13)
C17—C18—C19—Co3	−60.64 (19)	C1—Co1—Co3—Co2	−34.08 (7)
C17—C16—C20—C19	0.5 (3)	C8—Co1—Co3—Co2	−156.18 (9)
Co3—C16—C20—C19	−59.62 (18)	C9—Co1—Co3—Co2	−179.5 (2)
C17—C16—C20—Co3	60.12 (19)	C7—Co1—Co3—Co2	174.18 (8)
C18—C19—C20—C16	−0.5 (3)	C6—Co1—Co3—Co2	135.01 (8)
Co3—C19—C20—C16	60.31 (19)	C10—Co1—Co3—Co2	114.67 (11)
C18—C19—C20—Co3	−60.81 (18)	Co4—Co1—Co3—Co2	−69.732 (13)
C25—C21—C22—C23	0.0	C1—Co2—Co3—C19	−59.7 (7)
Co4—C21—C22—C23	−60.01 (7)	C11—Co2—Co3—C19	61.5 (7)
C25—C21—C22—Co4	60.01 (7)	C15—Co2—Co3—C19	72.7 (7)
C21—C22—C23—C24	0.0	C12—Co2—Co3—C19	146.6 (7)
Co4—C22—C23—C24	−60.30 (7)	C14—Co2—Co3—C19	109.6 (7)
C21—C22—C23—Co4	60.30 (7)	C13—Co2—Co3—C19	143.2 (7)
C22—C23—C24—C25	0.0	Co4—Co2—Co3—C19	−23.8 (7)
Co4—C23—C24—C25	−61.50 (7)	Co1—Co2—Co3—C19	−93.7 (7)
C22—C23—C24—Co4	61.50 (7)	C1—Co2—Co3—C18	−119.89 (14)
C23—C24—C25—C21	0.0	C11—Co2—Co3—C18	1.29 (17)
Co4—C24—C25—C21	−61.95 (7)	C15—Co2—Co3—C18	12.46 (15)
C23—C24—C25—Co4	61.95 (7)	C12—Co2—Co3—C18	86.34 (18)
C22—C21—C25—C24	0.0	C14—Co2—Co3—C18	49.40 (15)
Co4—C21—C25—C24	61.02 (7)	C13—Co2—Co3—C18	82.95 (14)
C22—C21—C25—Co4	−61.02 (7)	Co4—Co2—Co3—C18	−84.04 (13)
C2—C1—Co1—C8	−42.7 (2)	Co1—Co2—Co3—C18	−153.91 (13)
Co4—C1—Co1—C8	88.72 (10)	C1—Co2—Co3—C20	113.70 (17)
Co2—C1—Co1—C8	170.07 (9)	C11—Co2—Co3—C20	−125.1 (2)
C2—C1—Co1—C9	−3.2 (2)	C15—Co2—Co3—C20	−113.94 (18)
Co4—C1—Co1—C9	128.18 (9)	C12—Co2—Co3—C20	−40.1 (2)
Co2—C1—Co1—C9	−150.47 (9)	C14—Co2—Co3—C20	−77.00 (18)
C2—C1—Co1—C7	−62.4 (3)	C13—Co2—Co3—C20	−43.45 (17)
Co4—C1—Co1—C7	69.0 (2)	Co4—Co2—Co3—C20	149.56 (16)
Co2—C1—Co1—C7	150.33 (17)	Co1—Co2—Co3—C20	79.69 (16)
C2—C1—Co1—C6	39.9 (4)	C1—Co2—Co3—C17	−161.55 (13)
Co4—C1—Co1—C6	171.3 (3)	C11—Co2—Co3—C17	−40.37 (16)
Co2—C1—Co1—C6	−107.3 (3)	C15—Co2—Co3—C17	−29.20 (13)
C2—C1—Co1—C10	32.4 (3)	C12—Co2—Co3—C17	44.68 (17)
Co4—C1—Co1—C10	163.83 (9)	C14—Co2—Co3—C17	7.74 (13)
Co2—C1—Co1—C10	−114.82 (10)	C13—Co2—Co3—C17	41.29 (13)
C2—C1—Co1—Co4	−131.4 (2)	Co4—Co2—Co3—C17	−125.69 (11)
Co2—C1—Co1—Co4	81.35 (8)	Co1—Co2—Co3—C17	164.43 (11)
C2—C1—Co1—Co3	−172.4 (2)	C1—Co2—Co3—C16	153.75 (14)
Co4—C1—Co1—Co3	−40.97 (6)	C11—Co2—Co3—C16	−85.07 (16)
Co2—C1—Co1—Co3	40.38 (6)	C15—Co2—Co3—C16	−73.90 (14)
C2—C1—Co1—Co2	147.2 (3)	C12—Co2—Co3—C16	−0.02 (17)
Co4—C1—Co1—Co2	−81.35 (8)	C14—Co2—Co3—C16	−36.96 (14)
C7—C8—Co1—C1	−164.60 (15)	C13—Co2—Co3—C16	−3.41 (13)
C9—C8—Co1—C1	77.54 (16)	Co4—Co2—Co3—C16	−170.40 (12)
C7—C8—Co1—C9	117.9 (2)	Co1—Co2—Co3—C16	119.73 (12)
C9—C8—Co1—C7	−117.9 (2)	C1—Co2—Co3—Co4	−35.86 (6)
C7—C8—Co1—C6	37.53 (16)	C11—Co2—Co3—Co4	85.33 (11)
C9—C8—Co1—C6	−80.33 (17)	C15—Co2—Co3—Co4	96.50 (7)
C7—C8—Co1—C10	80.27 (17)	C12—Co2—Co3—Co4	170.38 (13)
C9—C8—Co1—C10	−37.58 (15)	C14—Co2—Co3—Co4	133.43 (7)
C7—C8—Co1—Co4	−112.65 (15)	C13—Co2—Co3—Co4	166.99 (6)
C9—C8—Co1—Co4	129.49 (14)	Co1—Co2—Co3—Co4	−69.871 (13)
C7—C8—Co1—Co3	−50.50 (18)	C1—Co2—Co3—Co1	34.01 (6)
C9—C8—Co1—Co3	−168.36 (12)	C11—Co2—Co3—Co1	155.20 (11)
C7—C8—Co1—Co2	−142.96 (19)	C15—Co2—Co3—Co1	166.37 (7)
C9—C8—Co1—Co2	99.2 (2)	C12—Co2—Co3—Co1	−119.75 (13)
C10—C9—Co1—C1	127.89 (17)	C14—Co2—Co3—Co1	−156.70 (7)
C8—C9—Co1—C1	−113.94 (16)	C13—Co2—Co3—Co1	−123.14 (6)
C10—C9—Co1—C8	−118.2 (2)	Co4—Co2—Co3—Co1	69.871 (13)
C10—C9—Co1—C7	−80.16 (18)	C2—C1—Co4—C24	−29.2 (2)
C8—C9—Co1—C7	38.01 (16)	Co1—C1—Co4—C24	−164.98 (8)
C10—C9—Co1—C6	−37.01 (17)	Co2—C1—Co4—C24	110.63 (8)
C8—C9—Co1—C6	81.16 (17)	C2—C1—Co4—C25	−51.2 (3)
C8—C9—Co1—C10	118.2 (2)	Co1—C1—Co4—C25	173.00 (10)
C10—C9—Co1—Co4	175.28 (13)	Co2—C1—Co4—C25	88.61 (14)
C8—C9—Co1—Co4	−66.55 (17)	C2—C1—Co4—C23	10.2 (2)
C10—C9—Co1—Co3	−87.0 (3)	Co1—C1—Co4—C23	−125.61 (8)
C8—C9—Co1—Co3	31.1 (3)	Co2—C1—Co4—C23	150.00 (8)
C10—C9—Co1—Co2	93.63 (17)	C2—C1—Co4—C21	31.7 (4)
C8—C9—Co1—Co2	−148.20 (13)	Co1—C1—Co4—C21	−104.1 (3)
C8—C7—Co1—C1	29.2 (3)	Co2—C1—Co4—C21	171.5 (3)
C6—C7—Co1—C1	147.59 (19)	C2—C1—Co4—C22	42.0 (2)
C6—C7—Co1—C8	118.4 (2)	Co1—C1—Co4—C22	−93.79 (10)
C8—C7—Co1—C9	−38.21 (15)	Co2—C1—Co4—C22	−178.18 (7)
C6—C7—Co1—C9	80.17 (17)	C2—C1—Co4—Co3	177.62 (19)
C8—C7—Co1—C6	−118.4 (2)	Co1—C1—Co4—Co3	41.81 (6)
C8—C7—Co1—C10	−81.35 (17)	Co2—C1—Co4—Co3	−42.59 (6)
C6—C7—Co1—C10	37.03 (16)	C2—C1—Co4—Co1	135.8 (2)
C8—C7—Co1—Co4	81.13 (16)	Co2—C1—Co4—Co1	−84.39 (8)
C6—C7—Co1—Co4	−160.49 (13)	C2—C1—Co4—Co2	−139.8 (2)
C8—C7—Co1—Co3	139.54 (15)	Co1—C1—Co4—Co2	84.39 (8)
C6—C7—Co1—Co3	−102.08 (15)	C23—C24—Co4—C1	83.61 (10)
C8—C7—Co1—Co2	151.14 (15)	C25—C24—Co4—C1	−158.74 (10)
C6—C7—Co1—Co2	−90.5 (2)	C23—C24—Co4—C25	−117.6
C10—C6—Co1—C1	−10.3 (4)	C25—C24—Co4—C23	117.6
C7—C6—Co1—C1	−129.3 (3)	C23—C24—Co4—C21	−80.0
C10—C6—Co1—C8	81.17 (17)	C25—C24—Co4—C21	37.7
C7—C6—Co1—C8	−37.88 (16)	C23—C24—Co4—C22	−37.3
C10—C6—Co1—C9	37.33 (16)	C25—C24—Co4—C22	80.3
C7—C6—Co1—C9	−81.72 (17)	C23—C24—Co4—Co3	−145.86 (11)
C10—C6—Co1—C7	119.0 (2)	C25—C24—Co4—Co3	−28.21 (10)
C7—C6—Co1—C10	−119.0 (2)	C23—C24—Co4—Co1	56.86 (12)
C10—C6—Co1—Co4	156.40 (13)	C25—C24—Co4—Co1	174.51 (13)
C7—C6—Co1—Co4	37.3 (2)	C23—C24—Co4—Co2	136.79 (6)
C10—C6—Co1—Co3	−157.47 (14)	C25—C24—Co4—Co2	−105.56 (6)
C7—C6—Co1—Co3	83.48 (15)	C24—C25—Co4—C1	34.52 (15)
C10—C6—Co1—Co2	−98.60 (16)	C21—C25—Co4—C1	152.31 (15)
C7—C6—Co1—Co2	142.36 (13)	C21—C25—Co4—C24	117.8
C6—C10—Co1—C1	175.86 (15)	C24—C25—Co4—C23	−37.9
C9—C10—Co1—C1	−65.05 (19)	C21—C25—Co4—C23	79.9
C6—C10—Co1—C8	−80.95 (17)	C24—C25—Co4—C21	−117.8
C9—C10—Co1—C8	38.14 (17)	C24—C25—Co4—C22	−80.6
C6—C10—Co1—C9	−119.1 (2)	C21—C25—Co4—C22	37.2
C6—C10—Co1—C7	−37.36 (16)	C24—C25—Co4—Co3	163.06 (7)
C9—C10—Co1—C7	81.73 (18)	C21—C25—Co4—Co3	−79.14 (7)
C9—C10—Co1—C6	119.1 (2)	C24—C25—Co4—Co1	−169.4 (3)
C6—C10—Co1—Co4	−133.3 (3)	C21—C25—Co4—Co1	−51.6 (2)
C9—C10—Co1—Co4	−14.2 (4)	C24—C25—Co4—Co2	96.41 (6)
C6—C10—Co1—Co3	32.8 (2)	C21—C25—Co4—Co2	−145.79 (6)
C9—C10—Co1—Co3	151.91 (13)	C24—C23—Co4—C1	−105.77 (10)
C6—C10—Co1—Co2	116.68 (15)	C22—C23—Co4—C1	136.04 (10)
C9—C10—Co1—Co2	−124.23 (15)	C22—C23—Co4—C24	−118.2
C2—C1—Co2—C11	22.2 (2)	C24—C23—Co4—C25	38.0
Co4—C1—Co2—C11	−111.34 (9)	C22—C23—Co4—C25	−80.2
Co1—C1—Co2—C11	167.77 (8)	C24—C23—Co4—C21	81.1
C2—C1—Co2—C15	55.9 (3)	C22—C23—Co4—C21	−37.1
Co4—C1—Co2—C15	−77.60 (12)	C24—C23—Co4—C22	118.2
Co1—C1—Co2—C15	−158.49 (8)	C24—C23—Co4—Co3	111.7 (2)
C2—C1—Co2—C12	−17.8 (2)	C22—C23—Co4—Co3	−6.5 (2)
Co4—C1—Co2—C12	−151.31 (7)	C24—C23—Co4—Co1	−153.83 (7)
Co1—C1—Co2—C12	127.80 (8)	C22—C23—Co4—Co1	87.98 (7)
C2—C1—Co2—C14	44.3 (5)	C24—C23—Co4—Co2	−69.86 (8)
Co4—C1—Co2—C14	−89.2 (4)	C22—C23—Co4—Co2	171.96 (8)
Co1—C1—Co2—C14	−170.1 (3)	C22—C21—Co4—C1	13.6 (3)
C2—C1—Co2—C13	−41.3 (3)	C25—C21—Co4—C1	−104.8 (3)
Co4—C1—Co2—C13	−174.84 (10)	C22—C21—Co4—C24	80.4
Co1—C1—Co2—C13	104.26 (13)	C25—C21—Co4—C24	−38.0
C2—C1—Co2—Co4	133.5 (2)	C22—C21—Co4—C25	118.4
Co1—C1—Co2—Co4	−80.89 (8)	C22—C21—Co4—C23	37.2
C2—C1—Co2—Co3	174.6 (2)	C25—C21—Co4—C23	−81.2
Co4—C1—Co2—Co3	41.10 (6)	C25—C21—Co4—C22	−118.4
Co1—C1—Co2—Co3	−39.79 (6)	C22—C21—Co4—Co3	−132.29 (7)
C2—C1—Co2—Co1	−145.6 (3)	C25—C21—Co4—Co3	109.27 (8)
Co4—C1—Co2—Co1	80.89 (8)	C22—C21—Co4—Co1	−75.60 (8)
C12—C11—Co2—C1	−106.61 (10)	C25—C21—Co4—Co1	165.96 (8)
C15—C11—Co2—C1	136.28 (10)	C22—C21—Co4—Co2	−178.29 (10)
C12—C11—Co2—C15	117.1	C25—C21—Co4—Co2	63.27 (9)
C15—C11—Co2—C12	−117.1	C21—C22—Co4—C1	−175.16 (11)
C12—C11—Co2—C14	79.2	C23—C22—Co4—C1	−56.47 (11)
C15—C11—Co2—C14	−37.9	C21—C22—Co4—C24	−81.0
C12—C11—Co2—C13	36.9	C23—C22—Co4—C24	37.7
C15—C11—Co2—C13	−80.2	C21—C22—Co4—C25	−37.5
C12—C11—Co2—Co4	−157.51 (7)	C23—C22—Co4—C25	81.2
C15—C11—Co2—Co4	85.38 (7)	C21—C22—Co4—C23	−118.7
C12—C11—Co2—Co3	133.74 (13)	C23—C22—Co4—C21	118.7
C15—C11—Co2—Co3	16.63 (13)	C21—C22—Co4—Co3	58.63 (8)
C12—C11—Co2—Co1	−89.13 (9)	C23—C22—Co4—Co3	177.32 (8)
C15—C11—Co2—Co1	153.76 (10)	C21—C22—Co4—Co1	127.88 (7)
C14—C15—Co2—C1	−175.56 (12)	C23—C22—Co4—Co1	−113.43 (7)
C11—C15—Co2—C1	−58.30 (11)	C21—C22—Co4—Co2	170.3 (6)
C14—C15—Co2—C11	−117.3	C23—C22—Co4—Co2	−71.1 (6)
C14—C15—Co2—C12	−79.1	C19—Co3—Co4—C1	−147.02 (11)
C11—C15—Co2—C12	38.2	C18—Co3—Co4—C1	170.68 (11)
C11—C15—Co2—C14	117.3	C20—Co3—Co4—C1	−114.43 (16)
C14—C15—Co2—C13	−36.8	C17—Co3—Co4—C1	133.71 (14)
C11—C15—Co2—C13	80.5	C16—Co3—Co4—C1	120.9 (6)
C14—C15—Co2—Co4	133.86 (7)	Co1—Co3—Co4—C1	−35.82 (7)
C11—C15—Co2—Co4	−108.88 (7)	Co2—Co3—Co4—C1	36.09 (7)
C14—C15—Co2—Co3	71.98 (7)	C19—Co3—Co4—C24	80.06 (13)
C11—C15—Co2—Co3	−170.76 (8)	C18—Co3—Co4—C24	37.75 (13)
C14—C15—Co2—Co1	115.4 (2)	C20—Co3—Co4—C24	112.64 (18)
C11—C15—Co2—Co1	−127.4 (2)	C17—Co3—Co4—C24	0.79 (15)
C11—C12—Co2—C1	84.38 (10)	C16—Co3—Co4—C24	−12.1 (6)
C13—C12—Co2—C1	−157.19 (10)	Co1—Co3—Co4—C24	−168.74 (10)
C13—C12—Co2—C11	118.4	Co2—Co3—Co4—C24	−96.83 (9)
C11—C12—Co2—C15	−38.7	C19—Co3—Co4—C25	61.81 (11)
C13—C12—Co2—C15	79.77 (5)	C18—Co3—Co4—C25	19.50 (11)
C11—C12—Co2—C14	−81.87 (5)	C20—Co3—Co4—C25	94.39 (16)
C13—C12—Co2—C14	36.6	C17—Co3—Co4—C25	−17.46 (13)
C11—C12—Co2—C13	−118.4	C16—Co3—Co4—C25	−30.3 (6)
C11—C12—Co2—Co4	41.03 (11)	Co1—Co3—Co4—C25	173.01 (6)
C13—C12—Co2—Co4	159.46 (12)	Co2—Co3—Co4—C25	−115.09 (6)
C11—C12—Co2—Co3	−123.41 (17)	C19—Co3—Co4—C23	−4.26 (19)
C13—C12—Co2—Co3	−4.98 (16)	C18—Co3—Co4—C23	−46.56 (19)
C11—C12—Co2—Co1	135.77 (8)	C20—Co3—Co4—C23	28.3 (2)
C13—C12—Co2—Co1	−105.80 (8)	C17—Co3—Co4—C23	−83.5 (2)
C15—C14—Co2—C1	14.7 (4)	C16—Co3—Co4—C23	−96.4 (6)
C13—C14—Co2—C1	−104.0 (4)	Co1—Co3—Co4—C23	106.94 (17)
C15—C14—Co2—C11	38.6	Co2—Co3—Co4—C23	178.85 (17)
C13—C14—Co2—C11	−80.08 (5)	C19—Co3—Co4—C21	23.43 (10)
C13—C14—Co2—C15	−118.7	C18—Co3—Co4—C21	−18.88 (11)
C15—C14—Co2—C12	82.06 (5)	C20—Co3—Co4—C21	56.01 (16)
C13—C14—Co2—C12	−36.6	C17—Co3—Co4—C21	−55.84 (13)
C15—C14—Co2—C13	118.7	C16—Co3—Co4—C21	−68.7 (6)
C15—C14—Co2—Co4	−62.94 (8)	Co1—Co3—Co4—C21	134.62 (6)
C13—C14—Co2—Co4	178.35 (8)	Co2—Co3—Co4—C21	−153.47 (6)
C15—C14—Co2—Co3	−115.70 (8)	C19—Co3—Co4—C22	−8.97 (11)
C13—C14—Co2—Co3	125.59 (8)	C18—Co3—Co4—C22	−51.27 (11)
C15—C14—Co2—Co1	−152.21 (9)	C20—Co3—Co4—C22	23.62 (16)
C13—C14—Co2—Co1	89.08 (9)	C17—Co3—Co4—C22	−88.23 (13)
C12—C13—Co2—C1	37.43 (16)	C16—Co3—Co4—C22	−101.1 (6)
C14—C13—Co2—C1	156.95 (16)	Co1—Co3—Co4—C22	102.23 (6)
C12—C13—Co2—C11	−37.8	Co2—Co3—Co4—C22	174.14 (6)
C14—C13—Co2—C11	81.68 (5)	C19—Co3—Co4—Co1	−111.20 (9)
C12—C13—Co2—C15	−81.92 (5)	C18—Co3—Co4—Co1	−153.50 (9)
C14—C13—Co2—C15	37.6	C20—Co3—Co4—Co1	−78.61 (15)
C14—C13—Co2—C12	119.5	C17—Co3—Co4—Co1	169.54 (12)
C12—C13—Co2—C14	−119.5	C16—Co3—Co4—Co1	156.7 (6)
C12—C13—Co2—Co4	−125.1 (3)	Co2—Co3—Co4—Co1	71.910 (14)
C14—C13—Co2—Co4	−5.6 (3)	C19—Co3—Co4—Co2	176.89 (9)
C12—C13—Co2—Co3	177.49 (8)	C18—Co3—Co4—Co2	134.59 (9)
C14—C13—Co2—Co3	−63.00 (8)	C20—Co3—Co4—Co2	−150.52 (15)
C12—C13—Co2—Co1	108.38 (8)	C17—Co3—Co4—Co2	97.63 (12)
C14—C13—Co2—Co1	−132.11 (8)	C16—Co3—Co4—Co2	84.8 (6)
C8—Co1—Co2—C1	−27.0 (2)	Co1—Co3—Co4—Co2	−71.910 (14)
C9—Co1—Co2—C1	47.74 (14)	C8—Co1—Co4—C1	−108.49 (12)
C7—Co1—Co2—C1	−145.4 (2)	C9—Co1—Co4—C1	−71.26 (13)
C6—Co1—Co2—C1	152.38 (14)	C7—Co1—Co4—C1	−148.95 (12)
C10—Co1—Co2—C1	100.67 (13)	C6—Co1—Co4—C1	−173.99 (17)
Co4—Co1—Co2—C1	−61.83 (9)	C10—Co1—Co4—C1	−60.3 (3)
Co3—Co1—Co2—C1	−132.01 (9)	Co3—Co1—Co4—C1	130.84 (9)
C1—Co1—Co2—C11	−23.12 (15)	Co2—Co1—Co4—C1	59.63 (9)
C8—Co1—Co2—C11	−50.1 (3)	C1—Co1—Co4—C24	34.09 (16)
C9—Co1—Co2—C11	24.62 (16)	C8—Co1—Co4—C24	−74.40 (16)
C7—Co1—Co2—C11	−168.5 (2)	C9—Co1—Co4—C24	−37.16 (17)
C6—Co1—Co2—C11	129.26 (17)	C7—Co1—Co4—C24	−114.86 (16)
C10—Co1—Co2—C11	77.55 (15)	C6—Co1—Co4—C24	−139.9 (2)
Co4—Co1—Co2—C11	−84.95 (12)	C10—Co1—Co4—C24	−26.2 (3)
Co3—Co1—Co2—C11	−155.13 (12)	Co3—Co1—Co4—C24	164.93 (13)
C1—Co1—Co2—C15	82.8 (3)	Co2—Co1—Co4—C24	93.73 (13)
C8—Co1—Co2—C15	55.8 (3)	C1—Co1—Co4—C25	−161.1 (3)
C9—Co1—Co2—C15	130.5 (3)	C8—Co1—Co4—C25	90.4 (3)
C7—Co1—Co2—C15	−62.6 (3)	C9—Co1—Co4—C25	127.6 (3)
C6—Co1—Co2—C15	−124.8 (3)	C7—Co1—Co4—C25	49.9 (3)
C10—Co1—Co2—C15	−176.5 (3)	C6—Co1—Co4—C25	24.9 (3)
Co4—Co1—Co2—C15	21.0 (3)	C10—Co1—Co4—C25	138.5 (4)
Co3—Co1—Co2—C15	−49.2 (3)	Co3—Co1—Co4—C25	−30.3 (2)
C1—Co1—Co2—C12	−79.69 (12)	Co2—Co1—Co4—C25	−101.5 (3)
C8—Co1—Co2—C12	−106.7 (2)	C1—Co1—Co4—C23	73.06 (11)
C9—Co1—Co2—C12	−31.96 (13)	C8—Co1—Co4—C23	−35.43 (11)
C7—Co1—Co2—C12	134.9 (2)	C9—Co1—Co4—C23	1.81 (12)
C6—Co1—Co2—C12	72.69 (14)	C7—Co1—Co4—C23	−75.89 (11)
C10—Co1—Co2—C12	20.98 (12)	C6—Co1—Co4—C23	−100.92 (16)
Co4—Co1—Co2—C12	−141.52 (8)	C10—Co1—Co4—C23	12.7 (3)
Co3—Co1—Co2—C12	148.29 (8)	Co3—Co1—Co4—C23	−156.10 (7)
C1—Co1—Co2—C14	175.79 (14)	Co2—Co1—Co4—C23	132.70 (7)
C8—Co1—Co2—C14	148.8 (2)	C1—Co1—Co4—C21	157.48 (12)
C9—Co1—Co2—C14	−136.47 (15)	C8—Co1—Co4—C21	48.99 (11)
C7—Co1—Co2—C14	30.4 (2)	C9—Co1—Co4—C21	86.23 (12)
C6—Co1—Co2—C14	−31.83 (16)	C7—Co1—Co4—C21	8.53 (11)
C10—Co1—Co2—C14	−83.53 (15)	C6—Co1—Co4—C21	−16.50 (17)
Co4—Co1—Co2—C14	113.96 (11)	C10—Co1—Co4—C21	97.2 (3)
Co3—Co1—Co2—C14	43.78 (11)	Co3—Co1—Co4—C21	−71.68 (8)
C1—Co1—Co2—C13	−130.61 (12)	Co2—Co1—Co4—C21	−142.88 (8)
C8—Co1—Co2—C13	−157.6 (2)	C1—Co1—Co4—C22	116.14 (11)
C9—Co1—Co2—C13	−82.87 (13)	C8—Co1—Co4—C22	7.65 (10)
C7—Co1—Co2—C13	84.0 (2)	C9—Co1—Co4—C22	44.89 (11)
C6—Co1—Co2—C13	21.77 (14)	C7—Co1—Co4—C22	−32.81 (10)
C10—Co1—Co2—C13	−29.94 (12)	C6—Co1—Co4—C22	−57.84 (16)
Co4—Co1—Co2—C13	167.56 (8)	C10—Co1—Co4—C22	55.8 (3)
Co3—Co1—Co2—C13	97.38 (8)	Co3—Co1—Co4—C22	−113.02 (6)
C1—Co1—Co2—Co4	61.83 (9)	Co2—Co1—Co4—C22	175.77 (6)
C8—Co1—Co2—Co4	34.8 (2)	C1—Co1—Co4—Co3	−130.84 (9)
C9—Co1—Co2—Co4	109.57 (11)	C8—Co1—Co4—Co3	120.67 (8)
C7—Co1—Co2—Co4	−83.55 (18)	C9—Co1—Co4—Co3	157.91 (10)
C6—Co1—Co2—Co4	−145.79 (12)	C7—Co1—Co4—Co3	80.21 (8)
C10—Co1—Co2—Co4	162.50 (10)	C6—Co1—Co4—Co3	55.17 (15)
Co3—Co1—Co2—Co4	−70.183 (14)	C10—Co1—Co4—Co3	168.8 (3)
C1—Co1—Co2—Co3	132.01 (9)	Co2—Co1—Co4—Co3	−71.208 (14)
C8—Co1—Co2—Co3	105.0 (2)	C1—Co1—Co4—Co2	−59.63 (9)
C9—Co1—Co2—Co3	179.75 (11)	C8—Co1—Co4—Co2	−168.12 (8)
C7—Co1—Co2—Co3	−13.36 (18)	C9—Co1—Co4—Co2	−130.89 (10)
C6—Co1—Co2—Co3	−75.61 (12)	C7—Co1—Co4—Co2	151.42 (8)
C10—Co1—Co2—Co3	−127.31 (10)	C6—Co1—Co4—Co2	126.38 (15)
Co4—Co1—Co2—Co3	70.183 (14)	C10—Co1—Co4—Co2	−120.0 (3)
C20—C19—Co3—C18	−118.2 (3)	Co3—Co1—Co4—Co2	71.208 (14)
C18—C19—Co3—C20	118.2 (3)	C11—Co2—Co4—C1	85.00 (11)
C18—C19—Co3—C17	37.56 (18)	C15—Co2—Co4—C1	126.35 (12)
C20—C19—Co3—C17	−80.6 (2)	C12—Co2—Co4—C1	58.05 (14)
C18—C19—Co3—C16	81.0 (2)	C14—Co2—Co4—C1	161.89 (12)
C20—C19—Co3—C16	−37.14 (18)	C13—Co2—Co4—C1	166.3 (3)
C18—C19—Co3—Co4	−89.81 (17)	Co3—Co2—Co4—C1	−130.21 (9)
C20—C19—Co3—Co4	152.01 (15)	Co1—Co2—Co4—C1	−59.92 (9)
C18—C19—Co3—Co1	−157.15 (15)	C1—Co2—Co4—C24	−90.56 (11)
C20—C19—Co3—Co1	84.67 (18)	C11—Co2—Co4—C24	−5.56 (11)
C18—C19—Co3—Co2	−67.4 (7)	C15—Co2—Co4—C24	35.79 (11)
C20—C19—Co3—Co2	174.4 (6)	C12—Co2—Co4—C24	−32.51 (13)
C17—C18—Co3—C19	118.5 (3)	C14—Co2—Co4—C24	71.34 (11)
C17—C18—Co3—C20	80.5 (2)	C13—Co2—Co4—C24	75.8 (3)
C19—C18—Co3—C20	−37.97 (17)	Co3—Co2—Co4—C24	139.24 (7)
C19—C18—Co3—C17	−118.5 (3)	Co1—Co2—Co4—C24	−150.48 (7)
C17—C18—Co3—C16	37.55 (19)	C1—Co2—Co4—C25	−135.17 (11)
C19—C18—Co3—C16	−80.92 (19)	C11—Co2—Co4—C25	−50.17 (11)
C17—C18—Co3—Co4	−135.47 (17)	C15—Co2—Co4—C25	−8.82 (11)
C19—C18—Co3—Co4	106.06 (16)	C12—Co2—Co4—C25	−77.12 (13)
C17—C18—Co3—Co1	166.62 (17)	C14—Co2—Co4—C25	26.72 (11)
C19—C18—Co3—Co1	48.2 (3)	C13—Co2—Co4—C25	31.2 (3)
C17—C18—Co3—Co2	−71.1 (2)	Co3—Co2—Co4—C25	94.62 (7)
C19—C18—Co3—Co2	170.43 (13)	Co1—Co2—Co4—C25	164.91 (7)
C16—C20—Co3—C19	−118.9 (3)	C1—Co2—Co4—C23	−49.17 (12)
C16—C20—Co3—C18	−81.0 (2)	C11—Co2—Co4—C23	35.83 (12)
C19—C20—Co3—C18	37.89 (17)	C15—Co2—Co4—C23	77.18 (12)
C16—C20—Co3—C17	−37.82 (19)	C12—Co2—Co4—C23	8.88 (14)
C19—C20—Co3—C17	81.12 (19)	C14—Co2—Co4—C23	112.73 (12)
C19—C20—Co3—C16	118.9 (3)	C13—Co2—Co4—C23	117.2 (3)
C16—C20—Co3—Co4	−169.07 (16)	Co3—Co2—Co4—C23	−179.37 (9)
C19—C20—Co3—Co4	−50.1 (2)	Co1—Co2—Co4—C23	−109.09 (9)
C16—C20—Co3—Co1	127.57 (17)	C1—Co2—Co4—C21	−175.45 (14)
C19—C20—Co3—Co1	−113.49 (16)	C11—Co2—Co4—C21	−90.45 (13)
C16—C20—Co3—Co2	62.3 (3)	C15—Co2—Co4—C21	−49.10 (13)
C19—C20—Co3—Co2	−178.74 (13)	C12—Co2—Co4—C21	−117.40 (15)
C18—C17—Co3—C19	−37.83 (17)	C14—Co2—Co4—C21	−13.56 (14)
C16—C17—Co3—C19	81.08 (19)	C13—Co2—Co4—C21	−9.1 (3)
C16—C17—Co3—C18	118.9 (3)	Co3—Co2—Co4—C21	54.34 (11)
C18—C17—Co3—C20	−81.51 (19)	Co1—Co2—Co4—C21	124.63 (11)
C16—C17—Co3—C20	37.40 (18)	C1—Co2—Co4—C22	15.7 (6)
C18—C17—Co3—C16	−118.9 (3)	C11—Co2—Co4—C22	100.7 (6)
C18—C17—Co3—Co4	64.0 (2)	C15—Co2—Co4—C22	142.0 (6)
C16—C17—Co3—Co4	−177.09 (15)	C12—Co2—Co4—C22	73.7 (6)
C18—C17—Co3—Co1	−155.0 (3)	C14—Co2—Co4—C22	177.6 (6)
C16—C17—Co3—Co1	−36.1 (5)	C13—Co2—Co4—C22	−178.0 (6)
C18—C17—Co3—Co2	134.65 (15)	Co3—Co2—Co4—C22	−114.5 (6)
C16—C17—Co3—Co2	−106.44 (17)	Co1—Co2—Co4—C22	−44.2 (6)
C20—C16—Co3—C19	37.68 (18)	C1—Co2—Co4—Co3	130.21 (9)
C17—C16—Co3—C19	−80.85 (19)	C11—Co2—Co4—Co3	−144.80 (8)
C20—C16—Co3—C18	81.20 (19)	C15—Co2—Co4—Co3	−103.45 (8)
C17—C16—Co3—C18	−37.34 (18)	C12—Co2—Co4—Co3	−171.74 (11)
C17—C16—Co3—C20	−118.5 (3)	C14—Co2—Co4—Co3	−67.90 (9)
C20—C16—Co3—C17	118.5 (3)	C13—Co2—Co4—Co3	−63.5 (3)
C20—C16—Co3—Co4	133.5 (6)	Co1—Co2—Co4—Co3	70.289 (14)
C17—C16—Co3—Co4	15.0 (7)	C1—Co2—Co4—Co1	59.92 (9)
C20—C16—Co3—Co1	−72.2 (2)	C11—Co2—Co4—Co1	144.92 (8)
C17—C16—Co3—Co1	169.27 (14)	C15—Co2—Co4—Co1	−173.73 (8)
C20—C16—Co3—Co2	−146.54 (15)	C12—Co2—Co4—Co1	117.97 (11)
C17—C16—Co3—Co2	94.93 (18)	C14—Co2—Co4—Co1	−138.19 (9)
C1—Co1—Co3—C19	137.85 (11)	C13—Co2—Co4—Co1	−133.7 (3)
C8—Co1—Co3—C19	15.74 (13)	Co3—Co2—Co4—Co1	−70.289 (14)
C9—Co1—Co3—C19	−7.6 (2)	C1—C2—Si1—C4	25.0 (2)
C7—Co1—Co3—C19	−13.89 (12)	C1—C2—Si1—C5	−99.4 (2)
C6—Co1—Co3—C19	−53.07 (12)	C1—C2—Si1—C3	144.8 (2)
C10—Co1—Co3—C19	−73.40 (14)		
